# Polymorphisms in the *SP110* and *TNF-α* Gene and Susceptibility to Pulmonary and Spinal Tuberculosis among Southern Chinese Population

**DOI:** 10.1155/2017/4590235

**Published:** 2017-12-21

**Authors:** Ying Zhou, Chun-yan Tan, Zhi-jiang Mo, Qi-le Gao, Dan He, Jiong Li, Rong-fu Huang, Yan-bing Li, Chao-feng Guo, Qiang Guo, Long-jie Wang, Guan-teng Yang, Hong-qi Zhang

**Affiliations:** ^1^Department of Laboratory Medicine, The People's Hospital of Guangxi Autonomous Region, Nanning, China; ^2^Department of Pharmacy, The People's Hospital of Guangxi Autonomous Region, Nanning, China; ^3^Department of Spine Surgery, Xiangya Spinal Surgery Center, Xiangya Hospital, Central South University, Changsha, China; ^4^Department of Neurology, The First Hospital of Changsha, Changsha, China; ^5^Department of Clinical Laboratory, The Second Affiliated Hospital, Fujian Medical University, Quanzhou, China; ^6^Department of Clinical Laboratory, Xiangya Hospital, Central South University, Changsha, China

## Abstract

**Objective:**

To investigate the association of single-nucleotide polymorphisms (SNPs) in *SP110* gene and *TNF-α* gene among pulmonary TB (PTB) and spinal TB (STB) patients.

**Methods:**

In a total of 190 PTB patients, 183 STB patients were enrolled as the case group and 362 healthy individuals at the same geographical region as the control group. The *SP110* SNPs (rs722555 and rs1135791) and the promoter -308G>A (rs1800629) and -238G>A (rs361525) polymorphisms in *TNF-α* were genotyped. *Results. TNF*-*α* -238G>A polymorphism was involved in susceptibility to STB, but not to PTB. The *TNF*-*α* -238 A allele was a protective factor against STB (A versus G: OR [95% CI] = 0.331 [0.113–0.972], *P* = 0.044). Furthermore, the presence of the -238 A allele was considered a trend to decrease the risk of STB (AG versus GG: *P* = 0.062, OR [95% CI] = 0.352 [0.118–1.053]; AA + AG versus GG: *P* = 0.050, OR [95CI%] = 0.335 [0.113–0.999]). However, *SP110* SNPs (rs722555 and rs1135791) and *TNF-α* -308G>A (rs1800629) showed no association with PTB and STB in all genetic models.

**Conclusion:**

The *TNF-α* -238 A allele appeared a protective effect against STB, whereas the *SP110* SNPs (rs722555 and rs1135791) and *TNF-α* -308G>A (rs1800629) showed no association with susceptibility to PTB and STB patients in southern China.

## 1. Introduction

Tuberculosis (TB), arising from *Mycobacterium* tuberculosis (MTB) infection, remains one of the leading causes of death in the world. The World Health Organization estimated that the TB incidence was 10.4 million, and there were approximately 1.7 million TB-related deaths that occurred among HIV-negative people (1.3 million) and HIV-positive individuals (374000) in 2016 [1]. Spinal TB (STB) is usually secondary to pulmonary lesions, accounting for 1%–5% TB and 15% of extrapulmonary TB (EPTB) worldwide [2, 3]. Host genetic factors have been shown to contribute to MTB infection outcomes.

The intracellular pathogen resistance 1 (*Ipr1*) gene, which is located in the *sst1* (super-susceptibility to TB 1) region, has an ability to mediate innate immunity in mouse TB models. The *Ipr1* gene may control the MTB growth by promoting macrophage activities and the apoptosis of infected macrophage [4, 5], and it has been shown to upregulate the expression of innate immunity gene to fight against TB [6]. *Speckled 110* (*SP110*) gene, the closest homology to the mouse *Ipr1* gene, is located on the human chromosome at 2q37.1 [4]. *SP110* is thought to be associated with susceptibility to TB [7, 8].

Tumor necrosis factor-*α* (TNF-*α*) has a key role in host resistance to TB infection. It is responsible not only for granuloma formation but also for granuloma integrity maintenance by restricting MTB growth within macrophages and preventing granuloma necrosis [9, 10]. An appropriate TNF-*α* production contributes to host defense against TB; however, deficient or excessive TNF-*α* might result in an unwanted immunopathological response. *TNF-α*-deficient mouse models have revealed an increased susceptibility to TB and rapid death, owing to the poorly formed granulomas, severe necrosis, and extensive dissemination of MTB [11]. In humans, patients receiving anti-TNF agent treatment have shown a higher risk of TB infection and reactivation [12–14].

Conversely, recent research has also confirmed that the overamplifying of TNF-*α* might be associated with increased severity of TB. Serum levels of TNF-*α* were significantly elevated in advanced TB than those in mild TB and healthy controls [15]. *SP110* may suppress the excessive activation of *TNF-α* by interacting with nuclear factor-*κ*B- (NF-*κ*B-) binding site in the *TNF-α* promoter region [16], leading to an appropriate milieu in response to TB infection.

The expression of TNF-*α* is mainly regulated by the gene promoter region. Single-nucleotide polymorphisms (SNPs) in the promoter of *TNF-α*, namely, -308G>A (rs1800629) and -238G>A (rs361525), have been reported to be associated with susceptibility to osteoarticular TB [17], while the role of these two polymorphisms in PTB is still debated [18–20].

Pulmonary TB (PTB) is mainly caused by respiratory tract infection. STB is usually secondary to lung infection whereas the patients with STB do not always have PTB. A previous study has reported that more than 50% of the STB patients are without pulmonary lesions [21]. Therefore, the anti-TB immune response in these two types of patients may vary which was due to the differential genetic factors [22]. In this study, we focused on the *SP110* (rs722555, rs1135791) and *TNF-α* (rs1800629, rs361525) polymorphisms among PTB patients, STB patients, and healthy controls from southern China. We aimed to investigate the influence and difference of *SP110* and *TNF-α* genetic variants on TB susceptibility.

## 2. Materials and Methods

### 2.1. Ethics Statement

This study had been conducted in accordance with the Declaration of Helsinki. All participants were briefed and consented to the study. The study was approved by the Ethical Committee of Central South University.

### 2.2. Subjects

A total of 190 patients with PTB, 183 patients with STB, and 362 healthy controls were included in this study. All subjects were from Southern China. All TB cases were diagnosed based on their clinical manifestations, radiographic findings, and laboratory examination. The diagnosis was confirmed microbiologically and/or histopathologically. Patients were followed up at least six months. All of them were recovered, and no recurrence was found until the final follow-up.

The study was performed in Xiangya Hospital, The People's Hospital of Guangxi Autonomous Region, The Second Affiliated Hospital of Fujian Medical University, and The First Hospital of Changsha from January 2009 to July 2016. This study was approved by the Ethical Committee of Central South University, China. Signed informed consent was obtained from all participants in accordance with the Helsinki Declaration.

### 2.3. Inclusion Criteria

(1) Patients with PTB are TB patients who were confirmed by demonstration of acid-fast bacilli in sputum smears, at least on two separate occasions and TB patients with only PTB lesions and no evidence of EPTB by imaging during the last follow-up. (2) Patients with STB had (i) clinical presentation—patients who had clinical features including chronic back or neck pain, fever, weakness, abscesses or sinus tracts, spinal localized tenderness or complications involving neurologic deficit, paraplegia, kyphosis, sensory disturbance, and bowel and bladder dysfunction; (ii) laboratory examination—patients with a positive T-SPOT TB test (Oxford Immunotec, Abingdon, UK) or increased C-reactive protein (CRP) and erythrocyte sedimentation rate (ESR); (iii) microbiological or histological examination—patients who underwent bone biopsy with or without biopsies of the paraspinal abscess and had a positive smear by Ziehl-Neelsen (ZN) staining or had a positive culture in Lowenstein-Jensen (L-J) medium, and pathohistological findings suggestive of TB involvement of the bone revealing granuloma formation, either caseating or noncaseating; and (iv) radiographic examination—patients who underwent MRI or CT scan that indicated spine involvement, such as bone destruction, disc space narrowing, with and without the presence of cold abscesses in adjacent muscle structures, and compression of the spinal cord or roots. (3) Healthy controls were healthy volunteers, who were matched according to age, gender, ethnicity, and region of origin with the included patients. Control subjects had no history of TB, no evidence of past exposure to TB, and no evidence of PTB and EPTB by imaging and had negative T-SPOT TB test (Oxford Immunotec, Abingdon, UK).

### 2.4. Exclusion Criteria

The inclusion criteria include individuals who had history of TB; individuals who had not received Bacillus Calmette-Guérin vaccination; individuals with disease impairing the immune system (diabetes, AIDS, infection, trauma, and tumor), autoimmune disease, or genetic disease; individuals using hormones or immune inhibitors; individuals who had positive T-SPOT TB test (Oxford Immunotec, Abingdon, UK); and individuals who died during follow-up period and had developed drug-resistant TB.

### 2.5. Genomic DNA Extraction

Two milliliters of peripheral blood was extracted for DNA extraction. Leukocyte genomic DNA was extracted using a Takara kit (Takara, Dalian, China) and according to the instructions. DNA was cryopreserved at −80°C.

### 2.6. Genotyping

The primers used for PCR and single-base extension were designed using the Mass Assay Designer 3.1 (Sequenom, San Diego, CA, USA), as shown in Table 1.

Multiplex polymerase chain reaction (PCR) was done in a 5 *μ*L amplification system in 384-well plates. This included 10 ng of genomic DNA, 0.5 *μ*L of 10x PCR Buffer (Sequenom, San Diego, CA, USA), 10 nmol of MgCl_2_, 2.5 nmol of deoxyribonucleoside triphosphate (dNTP) Mix (Takara, Dalian, China), 0.5 pmol of Primer Mix, 1 U HotStar Taq (Takara, Dalian, China), and 1.8 *μ*L of double-distilled water. Thermal cycling program was the following: 94°C for 15 minutes, 45 cycles of 94°C for 20 seconds, 56°C for 30 seconds, 72°C for 1 minute, and 72°C for 3 minutes.

To purify the PCR products, 0.5 U of shrimp alkaline phosphatase (SAP) enzyme (Fermentas, Ontario, Canada), 0.17 *μ*L of 0.24x SAP Buffer (Fermentas, Ontario, Canada), and 1.53 *μ*L of double-distilled water were added to each well. The mixture was incubated at 37°C for 40 minutes, followed by incubation at 85°C for 5 minutes.

The extension reaction was performed in a 9 *μ*L system; the reaction mix included 0.2 *μ*L of iPLEX Buffer Plus (Sequenom, San Diego, CA, USA), 0.2 *μ*L of iPLEX Termination Mix (Sequenom, San Diego, CA, USA), 0.94 *μ*L of iPLEX Extension Primer Mix, 0.041 *μ*L of iPLEX Enzyme (Sequenom, San Diego, CA, USA), 7 *μ*L of SAP and PCR reaction products, and 0.619 *μ*L of double-distilled water. The cycling conditions were as follows: 94°C for 30 seconds; 94°C for 5 seconds; 40 cycles of 52°C for 5 seconds; 5 cycles of 80°C for 5 seconds; and 72°C for 3 minutes. Purified extension reaction products were spotted onto SpectroCHIPs (Sequenom, San Diego, CA, USA) after removing salts with a cation-exchange resin. Genotyping was performed using the mass spectrometry platform (Sequenom, San Diego, CA, USA).

### 2.7. Statistical and Genetic Analysis

Analyses were done using SPSS software version 22.0 (SPSS Inc., Chicago, IL, USA). Firstly, a *t*-test was used to assess differences in age and gender between groups. Secondly, Hardy-Weinberg equilibrium (HWE) test of each polymorphism was performed using *χ*^2^ test, *P* < 0.05 means deviated from HWE. Thirdly, logistic regression analysis was used to test for the association between the polymorphisms and the TB (PTB/STB) risk. The frequency distributions of alleles (C2 versus C1), genotypes (C2C1 versus C1C1; C2C2 versus C1C1), the genetic dominant model (C2C2 + C2C1 versus C1C1), and recessive model (C2C2 versus C2C1 + C1C1) for each polymorphism were compared. The estimated odds ratios (ORs) and relative 95% confidence intervals (95% CIs) were adjusted for age and gender. Finally, linkage disequilibrium (LD) estimation analysis and haplotype association were performed using the SHEsis Online version (http://analysis.bio-x.cn/). For haplotype analysis, the lowest frequency threshold (LFT) was set to 0.03; that is, all single haplotypes with a frequency below this level were discarded in the following analysis. Fisher's exact test was used to find differences in haplotype frequency between TB (PTB/STB) patients and controls. Differences were considered statistically significant when *P* < 0.05. The changes were considered as trends when *P* ≥ 0.05 and *P* < 0.08.

## 3. Results

### 3.1. Characteristics of the Study Subjects

As shown in Table 2, a total of 190 PTB patients (male/female: 104/86; mean age: 41.55 ± 17.74 years), 183 STB patients (male/female: 95/88; mean age: 41.72 ± 18.03 years), and 362 healthy controls (male/female: 169/193; mean age: 45.11 ± 14.53 years) were included in this study.

### 3.2. Hardy-Weinberg Equilibrium (HWE) Test

The genotypic distributions of rs722555 and rs1135791 polymorphisms in the *SP110* gene accorded with HWE among PTB and STB patients and healthy controls (rs722555: *P*_PTB_ = 0.911, *P*_STB_ = 0.476, *P*_control_ = 0.912; rs1135791: *P*_PTB_ = 0.651, *P*_STB_ = 0.894, *P*_control_ = 0.461; data not shown).

The genotypic distributions of -308G>A and -238G>A polymorphisms in *TNF-α* gene accorded with HWE among study subjects (-308G>A: *P* = 0.681; -238G>A: *P* = 0.862; data not shown) and STB patients (-308G>A: *P*_PTB_ = 0.519, *P*_STB_ = 0.618, *P*_control_ = 0.087; -238G>A: *P*_PTB_ = 0.653, *P*_STB_ = 0.881, *P*_control_ = 0.235; data not shown).

### 3.3. Association of Polymorphisms in SP110 and TNF-*α* with PTB and STB

Spinal TB patients had a higher proportion of the *TNF-α* -238 A allele when compared with healthy controls (spinal TB versus controls: 1.1% versus 3.0%). Logistic regression analysis revealed that the -238 A allele was associated with STB after adjustment for age and gender (*P* = 0.044, Table 3). And the presence of -238 A allele also showed a trend to reduce the risk of STB (AG versus GG: *P* = 0.062; AA + AG versus GG: *P* = 0.050; Table 3).

However, the genotype and allele frequencies of *SP110* SNPs (rs722555, rs1135791) and *TNF-α* -308G>A (rs1800629) did not differ significantly between the PTB patients, STB patients, and healthy controls (Table 4).

### 3.4. Linkage Disequilibrium (LD) and Haplotype Analysis

LD analysis was carried out among the *SP110* (rs722555, rs1135791) and *TNF-α* (rs1800629, rs361525) polymorphisms. However, we found *SP110* and *TNF-α* SNPs were not in LD in both PTB control sample set (*SP110*: *r*^2^ = 0.201, *D*' = 0.881; *TNF-α*: *r*^2^ = 0.007, *D*' = 0.101) and STB control sample set (*SP110*: *r*^2^ = 0.223, *D*' = 0.918; *TNF-α*: *r*^2^ = 0.004, *D*' = 0.084). Thus, the haplotype analysis was not tested.

## 4. Discussion

*SP110* has been shown to promote macrophage activities and apoptosis and control the replication of MTB [4, 5], as well as upregulate innate immunity genes in mouse models infected with TB [6]. Furthermore, *SP110* has been identified an ability to reduce the TNF-*α* production by suppressing *TNF-α* gene promoter activity and upregulate antiapoptotic gene expression [16]. An appropriate expression of TNF-*α* not only contributes to the formation of granulomas, macrophage activities, and MTB intracellular killing but also accounts for the alleviated cell death and less severe necrosis lesions. All above are essential for TB defense and preventing unwanted immunopathology [9, 10]. Taken together, inappropriate expression of SP110 and TNF-*α* may lead to an impairment of the host immunity against TB, resulting in the dissemination of MTB.

The relationship between *SP110* polymorphisms and TB is mainly focused on PTB or all types of TB; however, the results exist controversial. Liang et al. [23] stated that individuals carrying rs1135791 C allele reduced the risk of TB disease (*P* < 0.0049; CT versus TT: OR [95% CI] = 0.61 [0.45–0.82]; CC versus TT: OR [95% CI] = 0.84 [0.36–2.00]) and the 1135791 C allele was protective against TB (*P* = 0.0062; C versus T: OR [95% CI] = 0.70 [0.54–0.91]). Moreover, Cai et al. [7] reported that the rs1135791 C allele was a protective factor of PTB in Chinese Han population (^∗∗^*P*_cor_ = 0.045; C versus T: OR [95% CI] = 0.0532 [0.57–0.89]), and further haplotype analysis showed that haplotypes of CGACCG (*P* = 5.00E − 06, OR [95% CI] = 0.44 [0.30–0.62]) and TGATTG (*P* = 2.59E − 04, OR [95% CI] = 3.52 [1.79–6.92]) in *SP110 rs1135791-rs3948464-rs1365776-rs9061-rs11556887-rs11679983* were related to TB risk. On the contrary, Cong et al. [8] revealed that the rs1135791 CT genotype (*P* < 0.0049; CT versus TT: OR [95% CI] = 1.9795 [1.1141–3.5169]) and rs722555 G allele (*P* = 0.0394; AG versus AA: OR [95% CI] = 1.7037 [0.7769–3.7364]; GG versus AA: OR [95% CI] = 3.0667 [1.2543–7.4978]) increased the risk of developing PTB in Chinese Han population. However, distinct races or populations might have different susceptible genetic factors. Furthermore, a wide-range geographical meta-analysis also suggested a negative association between *SP110* polymorphisms and TB risk among African, European descendant, and Asian mixed populations [24]. *SP110* SNP rs1135791 was shown to be related to PTB development in a certain Asian population, and the other SNP rs722555 was reported the associated with PTB in a Chinese Han population [7, 8, 23]. However, the genetic polymorphism difference whether it occurred in other populations especially in Chinese Han population remains unknown. Also, the genetic difference whether it existed between PTB and STB was unclear. Thus, these two important but not most widely studied SNPs were examined in our study. Our findings agreed with the stratified meta-analysis by ethnicity (African, European descendant, and Asian) [24]; none of our tested *SP110* SNPs showed a significant association with PTB and STB in all genetic models.

The promoter region of the *TNF-α* gene is highly polymorphic, and SNPs in the promoter may affect transcription and product expression [17, 25–27]. It has been reported that *TNF-α* promoter polymorphisms were related to TB risk [17, 18, 28]. Several meta-analyses showed a negative association [29, 30], whereas another has documented that the significant association with PTB is *TNF-α* -238G>A in the Asian population, while is *TNF-α* -308G>A in African population [31].

Controversial results might arise from various factors. Firstly, differential ethnic background leads to the different associations of the *TNF-α* SNPs with TB. A Thai study [32] reported that the *TNF-α* -308G>A and -238G>A polymorphisms were not associated with PTB risk, whereas an Iranian study [33] observed that the *-*308 A allele was a protective factor against PTB (*P* = 0.006, OR [95% CI] = 0.26 [0.07–0.77]). Secondly, predisposing polymorphisms differ in different types of TB. Lv et al. [17] have showed that the -308 GG genotype reduced osteoarticular TB (OA-TB) risk (*P* = 0.007, OR [95% CI] = 0.405 [0.147–0.657]). While GA genotype and A allele were risk factors of OA-TB in a Hebei population (GA: *P* = 0.003, OR [95% CI] = 3.112 [1.520–6.343]; A: *P* = 0.006, OR [95% CI] = 3.109 [1.676–6.538]), this was against the view of Merza et al. [33] who reported that the *-*308 A allele acted as a protective factor in PTB (A versus G: *P* = 0.006, OR = 0.26). Finally, the opposite association with the *TNF-α* SNPs existed in TB evolution, probably due to the consequence of natural selection. A previous study found that the -308 G allele (*P* = 0.02, OR = 1.8) and -238 A allele (*P* < 0.0001, OR = 2.2) represented susceptibility factors for TB, whereas haplotype of -308A-238G heterozygote showed a protective effect on TB (*P* = 0.01, OR [95% CI] = 0.46 [0.24–0.85]) [28].

In our study, we investigated the *TNF-α* -308G>A polymorphism (rs1800629) which was reported to be associated with the regulation of TNF-*α* levels [17, 27], and the other one *TNF-α* SNP was -238G>A (rs361525) which was TB-associated [18, 28]. Our results demonstrated that the *TNF*-*α* -238G>A was associated with STB, but not with PTB. The -238 A allele had shown a protective effect on STB (*P* = 0.044, OR [95% CI] = 0.331 [0.113–0.972]) after adjusting for age and gender. Furthermore, we observed a marginal statistically different distributions of AG genotype (AG versus GG: *P* = 0.062, OR [95CI%] = 0.352 [0.118–1.053]) and the dominant model (AA + AG versus GG: *P* = 0.050, OR [95CI%] = 0.335 [0.113–0.999], Table 3). Although the above two genetic models showed that the upper bound of 95% CI exceeded 1 (1.053) or closed to 1 (0.999), the presence of the -238 A allele was considered a trend to decrease the risk of STB. The regulatory role of TNF-*α* in the differentiation of osteoclast might be related to this [34]. However, we failed to find any association of *TNF-α* -308G>A polymorphism with PTB and STB in our study populations.

In conclusion, our study provides further evidence supporting the host genetic variability in TB susceptibility, especially in different types of TB infection. The *TNF-α* -238 A allele indicates a protective effect against STB, but not against PTB, whereas the *SP110* SNPs (rs722555 and rs1135791) and *TNF-α* -308G>A (rs1800629) show no association with susceptibility to PTB and STB in southern China. Our study may serve to assess the susceptible genetic factors and the possible outcomes of TB infection.

## Figures and Tables

**Table 1:** Primers sequences.

SNPs	Primer sequence (5′ → 3′)	Amplified fragment length (bp)
*SP110* rs722555	F: ACGTTGGATGAAGAGACATAGGGACAGGAG	120
	R: ACGTTGGATGTCCCCACTGTCTCATAAGTC	

*SP110* rs1135791	F: ACGTTGGATGGAAGGAAAAGGAAGGAACGC	103
	R: ACGTTGGATGAATACCTTCAGCAGCTCTCC	

*TNF-a* -308G>A	F: ACGTTGGATGGGTCCCCAAAAGAAATGGAG	100
(rs1800629)	R: ACGTTGGATGGATTTGTGTGTAGGACCCTG	

*TNF-a* -238G>A	F: ACGTTGGATGCACACAAATCAGTCAGTGGC	101
(rs361525)	R: ACGTTGGATGATCAAGGATACCCCTCACAC	

SNP: single-nucleotide polymorphism; PCR: polymerase chain reaction; F: forward; R: reverse.

**Table 2:** Age and sex characteristics of patients with PTB, patients with STB, and controls.

	PTB (*n* = 190)	STB (*n* = 183)	Controls (*n* = 362)
Age (years ± SD)	41.55 ± 17.74	41.72 ± 18.03	45.11 ± 14.53
Sex (male/female)	104/86 (0.55/0.45)	95/88 (0.52/0.48)	169/193 (0.47/0.53)

PTB: pulmonary tuberculosis; STB: spinal tuberculosis.

**Table 3:** The frequencies of polymorphisms in *TNF-α* gene and association analyses with the risk of PTB and STB.

SNPs	Genotype /allele	PTB (%)	STB (%)	Controls (%)	PTB versus controls		STB versus controls		STB versus PTB	
		(*n* = 190)	(*n* = 183)	(*n* = 362)	*P* ^a^	OR [95% CI]^a^	*P* ^a^	OR [95% CI]^a^	*P* ^a^	OR [95% CI]^a^
*TNF-a* -308G>A (rs1800629)										
Genotype	AA	0 (0)	0 (0)	2 (0.6)		NA		NA		NA
	AG	17 (8.9)	13 (7.1)	27 (7.5)	0.673	1.151 [0.599–2.211]	0.840	0.931 [0.468–1.854]	0.660	0.841 [0.389–1.818]
	GG	173 (91.1)	170 (92.9)	333 (92.0)		Ref		Ref		Ref

Allele	A	17 (4.5)	13 (3.6)	31 (4.3)	1.000	1.000 [0.537–1.863]	0.547	0.816 [0.421–1.581]	0.540	0.789 [0.370–1.684]
	G	363 (95.5)	353 (96.4)	693 (95.7)		Ref		Ref		Ref

Dominant model	AA + AG	17 (8.9)	13 (7.1)	29 (8.0)	0.840	1.069 [0.561–2.037]	0.677	0.865 [0.438–1.710]	0.660	0.841 [0.389–1.818]
	GG	173 (91.1)	170 (92.9)	333 (92.0)		Ref		Ref		Ref

Recessive model	AA	0 (0)	0 (0)	2 (0.6)		NA		NA		NA
	AG + GG	190 (100.0)	183 (100.0)	360 (99.4)		Ref		Ref		Ref

*TNF-a* -238G>A (rs361525)										
Genotype	AA	0 (0)	0 (0)	1 (0.3)		NA		NA		NA
	AG	12 (6.3)	4 (2.2)	20 (5.5)	0.842	0.925 [0.430–1.987]	0.062^∗^	0.352 [0.118–1.053]	0.140	0.414 [0.128–1.337]
	GG	178 (93.7)	179 (97.8)	341 (94.2)		Ref		Ref		Ref

Allele	A	12 (3.2)	4 (1.1)	22 (3.0)	0.589	0.817 [0.392–1.701]	0.044^†^	0.331 [0.113–0.972]	0.182	0.456 [0.144–1.445]
	G	368 (96.8)	362 (98.9)	702 (97.0)		Ref		Ref		Ref

Dominant model	AA + AG	12 (6.3)	4 (2.2)	21 (5.8)	0.725	0.873 [0.409–1.861]	0.050^∗^	0.335 [0.113–0.999]	0.140	0.414 [0.128–1.337]
	GG	178 (93.7)	179 (97.8)	341 (94.2)		Ref		Ref		Ref

Recessive model	AA	0 (0)	0 (0)	1 (0.3)		NA		NA		NA
	AG + GG	190 (100.0)	183 (100.0)	361 (99.7)		Ref		Ref		Ref

PTB: pulmonary tuberculosis; STB: spinal tuberculosis; OR: odds ratio; CI: confidence interval; Ref: reference; SNP: single-nucleotide polymorphism; NA: not calculated as zero count in at least one cell of the two by two table. ^a^The estimated odds ratios (ORs) and relative 95% confidence intervals (95% CI) were adjusted for age and gender. ^∗^*P* ≥ 0.05 and *P* < 0.08; the changes were considered as trends. ^†^*P* < 0.05.

**Table 4:** The frequencies of polymorphisms in *SP110* gene and association analyses with the risk of PTB and STB.

SNPs	Genotype /Allele	PTB (%)	STB (%)	Controls (%)	PTB versus Controls		STB versus Controls		STB versus PTB	
		(*n* = 190)	(*n* = 183)	(*n* = 362)	*P* ^a^	OR [95% CI]^a^	*P* ^a^	OR [95% CI]^a^	*P* ^a^	OR [95% CI]^a^
*SP110* rs722555										
Genotype	AA	39 (20.5)	28 (15.3)	60 (16.6)	0.245	1.364 [0.808–2.305]	0.784	0.927 [0.538–1.597]	0.235	0.688 [0.372–1.276]
	AG	95 (50.0)	93 (50.8)	176 (48.6)	0.403	1.191 [0.790–1.797]	0.743	1.068 [0.719–1.586]	0.690	0.909 [0.568–1.453]
	GG	56 (29.5)	62 (33.9)	126 (34.8)		Ref		Ref		Ref

Allele	A	173 (45.5)	149 (40.7)	296 (40.9)	0.227	1.171 [0.906–1.514]	0.894	0.983 [0.760–1.270]	0.261	0.844 [0.628–1.135]
	G	207 (54.5)	217 (59.3)	428 (59.1)		Ref		Ref		Ref

Dominant model	AA + AG	134 (70.5)	121 (66.1)	236 (65.2)	0.282	1.237 [0.839–1.825]	0.868	1.032 [0.709–1.503]	0.461	0.845 [0.542–1.321]
	GG	56 (29.5)	62 (33.9)	126 (34.8)		Ref		Ref		Ref

Recessive model	AA	39 (20.5)	28 (15.3)	60 (16.6)	0.384	1.227 [0.775–1.943]	0.646	0.891 [0.546–1.455]	0.258	0.730 [0.424–1.259]
	AG + GG	151 (79.5)	155 (84.7)	302 (83.4)		Ref		Ref		Ref

*SP110* rs1135791										
Genotype	CC	7 (3.7)	5 (2.7)	10 (2.8)	0.683	1.235 [0.449–3.397]	0.958	0.971 [0.324–2.913]	0.614	0.735 [0.223–2.427]
	CT	54 (28.4)	49 (26.8)	89 (24.6)	0.205	1.303 [0.866–1.960]	0.532	1.139 [0.757–1.714]	0.635	0.893 [0.561–1.425]
	TT	129 (67.9)	129 (70.5)	263 (72.7)		Ref		Ref		Ref

Allele	C	68 (17.9)	59 (16.1)	109 (15.1)	0.219	1.238 [0.881–1.740]	0.644	1.085 [0.768–1.533]	0.506	0.876 [0.593–1.294]
	T	312 (82.1)	307 (83.9)	615 (84.9)		Ref		Ref		Ref

Dominant model	CC + CT	61 (32.1)	54 (29.5)	99 (27.3)	0.194	1.296 [0.876–1.919]	0.568	1.122 [0.757–1.663]	0.561	0.876 [0.559–1.372]
	TT	129 (67.9)	129 (70.5)	263 (72.7)		Ref		Ref		Ref

Recessive model	CC	7 (3.7)	5 (2.7)	10 (2.8)	0.785	1.150 [0.421–3.145]	0.910	0.939 [0.315–2.801]	0.649	0.759 [0.232–2.488]
	CT + TT	183 (96.3)	178 (97.3)	352 (97.2)		Ref		Ref		Ref

PTB: pulmonary tuberculosis; STB: spinal tuberculosis; OR: odds ratio; CI: confidence interval; Ref: reference; SNP: single-nucleotide polymorphism. ^a^The estimated odds ratios (ORs) and relative 95% confidence intervals (95% CI) were adjusted for age and gender.

## Additional Points

*Highlight.* (i) SP110 is responsible for host innate immunity in tuberculosis (TB) controlling. Tumor necrosis factor-*α* (TNF-*α*) contributes to the protective immune response against TB. (ii) Interaction of *SP110* and *TNF-α* may induce a downregulation of TNF-*α*, leading to an appropriate milieu in response to TB infection. (iii) The *TNF-α* -238 A allele appeared to have a protective effect against STB, whereas the *SP110* (rs722555, rs1135791) and *TNF-α* -308G>A (rs1800629) polymorphisms were not associated with susceptibility to PTB and STB patients in southern China.
